# (+)-Borneol inhibits neuroinflammation and M1 phenotype polarization of microglia in epileptogenesis through the TLR4-NFκB signaling pathway

**DOI:** 10.3389/fnins.2024.1497102

**Published:** 2024-11-13

**Authors:** Shuo Li, Alhamdu Adamu, Yucai Ye, Fankai Gao, Rulin Mi, Guofang Xue, Zhaojun Wang

**Affiliations:** ^1^Second Clinical Medical School, Shanxi Medical University, Taiyuan, China; ^2^Department of Physiology, Shanxi Medical University, Taiyuan, China; ^3^Department of Neurology, Second Hospital of Shanxi Medical University, Taiyuan, China

**Keywords:** epileptogenesis, (+)‐borneol, microglia, neuroinflammation, TLR4‐NFκB

## Abstract

**Objective:**

To investigate the effect of (+)-borneol on neuroinflammation and microglia phenotype polarization in epileptogenesis and its possible mechanism.

**Methods:**

Based on mouse models of status epilepticus (SE) induced by pilocarpine, and treated with 15 mg/kg (+)-borneol, western-blot was used to detect the expressions of NeuN, Iba-1, TLR4, p65 and p-p65 in the hippocampus. Immunofluorescence was used to detect the expression of apoptosis-related proteins Bax and Bcl-2. To explore the effect of (+)-borneol on microglia *in vitro*, we used the kainic acid-induced microglia model and the concentration of (+)-borneol was 25 μM according to CCK-8 results. The levels of tumor necrosis factor-*α* (TNF-α), interleukin-1β (IL-1β) and interleukin-10 (IL-10) in the supernatant of each group was detected by ELISA. The nitric oxide (NO) content in the supernatant was detected by Griess method. The expressions of Iba-1 and TLR4-NFκB signaling pathway-related proteins (TLR4, p65, p-p65) were detected by Western-Blot. Immunofluorescence was used to detect microglia’s M1 and M2 phenotype polarization and the expression of Iba-1 and TLR4.

**Results:**

(+)-borneol reduced hippocampal neuronal injury, apoptosis, and microglia activation by inhibiting the TLR-NFκB signaling pathway in SE mice. TLR4 agonist LPS partially reversed the neuroprotective effect of (+)-borneol. In the KA-induced microglia model, (+)-borneol inhibited microglia activation, M1 phenotype polarization, and secretion of pro-inflammatory cytokines through the TLR4-NFκB signaling pathway. LPS treatment inhibited the therapeutic effects of (+)-borneol.

**Conclusion:**

(+)-borneol inhibits microglial neuroinflammation and M1 phenotype polarization through TLR4-NFκB signaling pathway and reduces neuronal damage and apoptosis in SE mice. Therefore, (+)-borneol may be a potential drug for epilepsy modification therapy.

## 1 Introduction

Epilepsy is a chronic brain disease characterized by recurrent seizures (Beghi, 2020). The causes of the epilepsy mainly include malformtion of cortical development, cerebrovascular diseases, brain tumors, traumatic brain injury, and central nervous system infection (Pitkänen et al., 2015). At present, epilepsy has become the second most common disease of the nervous system, affecting about 70 million people around the world (Thijs et al., 2019). Comorbidities such as anxiety, depression and stigma bring great pressure on patients and a heavy burden to families and society (Trinka et al., 2019; Beghi, 2020). Antiseizure medications (ASMs) are currently the main means of controlling seizures, such as sodiumvalproate, lamotrigine, levetiracetam, carbamazepine (Manford, 2017). However, they can only reduce the frequency and/or severity of seizures, cannot interfere with the process of the disease or change the prognosis and one-third of patients are resistant to existing drugs (Thijs et al., 2019).

Disease-modifying therapy (DMT) is a treatment that modifies the course of disease through medical intervention, which has been widely used in various neurological diseases (Chiò et al., 2020; Liu et al., 2021a; Tabrizi et al., 2022). In the existing studies on epilepsy modifying therapy, the frequency and duration of hippocampal paroxysmal discharges in epileptic mice can be reduced by inhibiting neuroinflammation (Gong et al., 2022), or the comorbidities such as learning and memory deficits of rats post-status epilepticus (SE) can be improved (Casillas-Espinosa et al., 2023). Both of them can achieve the purpose of disease modification. It has become the main research direction of epilepsy to search for new therapeutic targets and more effective drugs to delay the development of epilepsy, improve the pathological damage after seizures, and achieve the purpose of epilepsy modification therapy (Galanopoulou et al., 2021).

Epileptogenesis refers to the chronic process of the formation of epileptic pathological foci (Pitkänen et al., 2015).In other words, the brain undergoes molecular cellular changes caused by epileptogenic factors, then the excitability of neurons increases and leads to spontaneous recurrent seizures (SRS) (Pitkänen et al., 2007). The whole process is called epileptogenesis, which is the main target of epilepsy modification therapy. In this process, a series of pathophysiological changes occur, including abnormal neuroinflammation, excessive activation of glia, massive secretion of inflammatory factors, active plasticity of abnormal neuronal networks and dendritic spines, dysregulation of voltage-gated ion channels, neuronal damage, apoptosis, blood–brain barrier damage, and infiltration of peripheral immune cells into the brain (Li et al., 2023; Jean et al., 2023; Issa et al., 2023). Recent studies have shown that neuroinflammation and its coupled activation of glia and release of inflammatory factors are the initiating factors of epilepsy and run through the whole process (Mukhtar, 2020; Soltani Khaboushan et al., 2022).Neuroinflammation and its related pathways have become important targets for drug development, and drugs targeting neuroinflammation are also considered promising drugs and put into basic or clinical research (Clossen and Reddy, 2017).

Microglia are the resident immune cells in the nervous system, which are involved in maintaining the homeostasis of the central nervous system (Borst et al., 2021). According to the different proinflammatory or anti-inflammatory functions, microglia are generally divided into M1 phenotype (neurotoxic) and M2 phenotype (neuroprotective), and the two phenotypes can transform into each other (Kwon and Koh, 2020). In the early stage of epilepsy, microglia can be activated and respond to neuroinflammation first (Sano et al., 2021). and the M1 phenotype is significantly increased, accompanied by a large number of inflammatory factors (Benson et al., 2015). Activated microglia are involved in subsequent astrocyte activation and can contribute to astrocyte dysfunction and acute seizures (Henning et al., 2023). A variety of drugs can inhibit neuroinflammation by regulating the phenotype polarization of microglia (Yang et al., 2017; Zhang et al., 2019; Liu et al., 2021b). In addition to participating in neuroinflammation to increase neuronal excitability, microglia can also cause non-inflammatory changes through enhanced mTOR signaling, which disrupts central nervous system homeostasis and eventually leads to SRS (Zhao et al., 2018). The inhibition of microglia after SE reduced the frequency of SRS, the duration and severity of seizures (Wang et al., 2015). Therefore, microglia play an important role in epileptogenesis and may be a potential target for epilepsy modification therapy.

(+)-Borneol (C10H18O) is the main component of natural borneol. It is believed to have the functions of “medicinal guide” (Ren et al., 2024) and “clearing heat” (Zou et al., 2017; Zhong et al., 2023) in traditional Chinese medicine, so it is widely used in prescriptions for the treatment of mental disorders. Recent studies have shown that (+)-borneol can inhibit oxidative stress (Hua et al., 2021; Chen et al., 2022), neuroinflammation (Liu et al., 2011) and apoptosis (Liu et al., 2011), and it can cross the blood–brain barrier (Liu et al., 2021) to play a neuroprotective role in a variety of nervous system diseases (Wang et al., 2019; Xie et al., 2022; Ma et al., 2023). Our previous studies have shown that (+)-borneol can inhibit neuroinflammation and apoptosis in the hippocampus of rats with status epilepticus (SE) (Gao et al., 2023). However, the effects of (+)-borneol on neuroinflammation and phenotype polarization of microglia in epilepsy are still unclear. In the present study, we examined the expression of inflammatory cytokines, microglia phenotypic markers, neuronal markers, apoptosis-related proteins and TLR4-NFκB signaling pathway-related proteins to evaluate whether (+)-borneol could play a neuroprotective role by regulating microglial neuroinflammation and phenotypic polarization *in vitro* and *in vivo* through TLR4-NFκB signaling pathway.

## 2 Methods

### 2.1 Chemicals

(+)-Borneol was gifted to Simcere Pharmaceuticals. LPS was purchased from Beijing Solarbio Science & Technology Co., Ltd. (Cat No:L8880). Lithium chloride was purchased from Sigam (United States). Atropine and Pilocarpine was purchased from Shanghai Aladdin Biochemical Technology Co., Ltd. Diazepam was purchased from Jinyao Pharmaceutical. KA was purchased from Yuanye (Shanghai, China, Cat No:S30773).

### 2.2 Animals

C57BL/6 male mice, aged 8 weeks, 18-22 g, were purchased from the Animal Center of Shanxi Medical University and raised in the tertiary SPF animal facility of the Animal Experimental Center of Shanxi Medical University. The average room temperature was 20–25°C, the relative humidity was 30–50%, and the light and dark cycles were alternating for 12 h. They had free access to water and food. After 1 week of acclimatization, the mice were randomly divided into 4 groups: normal Control group (Control group), epilepsy model group (SE group), (+)-borneol group (SE + Borneol group), and TLR4 agonist group (SE + Borneol+LPS group), with 12 mice in each group. All experimental protocols were in accordance with NIH guidelines and have been reviewed by the Ethics Committee of the Second Hospital of Shanxi Medical University. The ethics record number was DW2023018.

### 2.3 Preparation of (+)-borneol solution

(+)-Borneol was dissolved in dimethyl sulfoxide (DMSO) in 100 mg/mL, diluted to 2 mg/mL in normal saline for intraperitoneal injection *in vivo*, or diluted to 1 mM solution in PBS for cell experiments.

### 2.4 Animal model preparation and grouping treatment

The mice in the SE group, SE + Borneol group and SE + Borneol+LPS group were intraperitoneally injected with lithium chloride (127 mg/kg) for pretreatment and were intraperitoneally injected with atropine (1 mg/kg) 20 h later. Pilocarpine (200 mg/kg) was injected intraperitoneally 30 min later. About 20–30 min later, seizures were observed. Thirty minutes after successful modeling, diazepam (10 mg/kg) was injected intraperitoneally to terminate the seizure. The mice in the Control group were intraperitoneally injected with the same volume of normal saline. Animal behavior was assessed according to the Racine grade (Racine, 1972). The criteria for successful modeling were Racine grade IV (generalized tonic–clonic standing on the hind limbs) or grade V (generalized tonic–clonic standing even unable to maintain balance) lasting more than 30 min, or failure to restore normal behavior between seizures.

The mice in the SE + Borneol group were intraperitoneally injected with (+)-borneol solution (15 mg/kg) daily for 7 days, and those in the SE + Borneol+LPS group were intraperitoneally injected with LPS solution (0.5 mg/kg) 6 h before (+)-borneol injection. The other groups were injected with the same volume of solvent at the same time point.

### 2.5 Immunofluorescence

After 7 days of drug administration, brains were removed by heart perfusion, fixed and dehydrated, snap-frozen in liquid nitrogen, and stored in a cryogenic refrigerator at −80°C. Frozen sections were 20 μm brain slices. After 1 h of equilibrium at room temperature, the surrounding embedding agent was washed off with PBS and fixed with 4% paraformaldehyde. Brain slices were fixed with 4% paraformaldehyde for 30 min at room temperature, incubated with 0.5%TritonX-100 for 15 min at room temperature, blocked with BSA for 1 h, and incubated with the next primary antibody drop at 4°C overnight: Bax (1:50, Bioworld, USA), Bcl-2 (1:50, Bioworld, USA). The next day, after rewarming at room temperature for 1 h, fluorescent secondary antibody (1:150, Bioss, China) was incubated in the dark for 1 h, and DAPI was incubated in the dark for 10 min after washing. Anti-fluorescence quencher was added to seal the slides, and the slides were observed under the fluorescence microscope (Olympus, BX51, Japan) and photographed under 10x objective lens with the same parameters as the control group. Finally, Image-J was used for analysis.

### 2.6 Cell culture

The BV2 microglia cell line was purchased from Wuhan Pricella Biotechnology Co., LTD.

BV2 microglia cells were cultured in the complete medium (DMEM/F12, 10% fetal bovine serum, 1% penicillin–streptomycin solution) at 37°C and 5%CO2 incubator (Thermo, Germany). The culture medium was discarded, washed twice with phosphate-buffered saline (PBS), and digested with 0.25% trypsin in the incubator for 5 min. The digestion was terminated with 2 times the amount of trypsin in complete medium, centrifuged at 1000 rpm for 5 min, and the supernatant was discarded and the complete medium was resuspended and passaged at a ratio of 1:3.

### 2.7 CCK-8

Cells in the logarithmic phase were collected and seeded in 96-well plates at 50000 /mL cell concentration and 100 μL per well. The cells were treated with 600 μM KA (Source, Shanghai, China). After 12 h, (+)-borneol solution of 6 μM, 12.5 μM, 25 μM, 50 μM, 100 μM and 200 μM was added, respectively. After 12 h, the culture medium was discarded, and the culture medium containing 10 μL CCK-8 solution was added to each well. After incubation at 37°C for 1 h, the absorbance was measured by the microplate reader (Bio-Tek, United States) at 450 nm. Cell survival rate = [(experimental well-blank well)/ (control well-blank well)] × 100%. The CCK-8 kit was purchased from Boster, China (Cat No:AR1160).

### 2.8 Groups and treatment of cells

Microglia were seeded in 6-well or 12-well plates at a density of 50,000 cells /ml. The cells were divided into 4 groups: Control group (Control group), model group (KA group), (+)-borneol group (KA + Borneol group), and TLR4 agonist group (KA + Borneol+LPS group). After 24 h, the cells adhered to the culture dishes, the medium of each group was discarded and the cells were washed twice with PBS. The complete medium containing 600 μM KA was added in the KA group, KA + Borneol group and KA + Borneol+LPS group. In the KA + Borneol group and KA + TLR4 agonist group, (+)-borneol solution with a final concentration of 25 μM was added after 12 h of KA treatment. In the KA + Borneol+LPS group, LPS solution with a final concentration of 50 ng/mL was added 1 h before (+)-borneol was added.

### 2.9 Elisa

After the cells were grouped and cultured, the cell culture supernatant of each group was collected and centrifuged at 1000 rpm for 5 min, and the supernatant was removed and placed at −20°C for later use, which were tested within 1 week of collection According to the instructions of ELISA, the absorbance was measured at 450 nm using a microplate reader and the contents of TNF-*α* (Boster, China), IL-1β (Boster, China) and IL-10 (Bioswamp, China) in the cell supernatant of each group were calculated.

### 2.10 Griess

Using Griess kit (Beyotime, S0021S, China), Griess Reagent I and II reagents in the kit were equilibrated to room temperature. According to the instructions, the complete medium same as the cell culture was used to dilute standards (0, 1, 2, 5, 10, 20, 40, 60, 100 μM). In a 96-well plate, 50 μL of standard or cell supernatant from each group was added to each well. 50 μL of Griess Reagent I and II reagents that had equilibrated to room temperature were successively added to each well. Stir gently and mix well. The absorbance was measured at 540 nm using a microplate reader. The concentration of nitric oxide in the supernatant of each group was calculated by drawing a standard curve based on the results of the standard.

### 2.11 Western blot

After 7 days of drug administration, the animals were sacrificed by neck removal, and the hippocampus was removed from the whole brain, weighed, cut into pieces on ice, and thoroughly blown after adding precooled RIPA (the ratio of tissue weight and RIPA was 1 g:10 mL) and 1% protease inhibitors. The tissues were crushed by an ultrasonic morcellator (QSONICA, United States) for 2 s ultrasound and 2 s intervals. The time of morcellation was determined according to the situation of the tissue. Then the tissue was centrifuged at 13000 rpm for 25 min at 4°C, and the protein concentration was determined by BCA method and quantified to 35 μg/10 μL. The appropriate concentration of separation gel was selected according to the molecular weight, and the proteins were separated by electrophoresis and transferred to the PVDF membrane. The protein was blocked with protein-free rapid blocking solution (Boster, China) at room temperature for 15 min, and incubated in the following primary antibody solution at 4°C overnight: NeuN (1:500, Abclonal, China), Iba-1 (1:1000, Bioworld, United States), TLR4 (1:1000, Proteintech, China), p65 (1:2000, Bioss, China), p-p65 (1:1000, Bioss, China), GAPDH (1:1000, Bioworld, United States), *β*-actin (1:1000, Bioworld, China). Secondary antibody (1:10000, Servicebio, China) incubation and exposure were performed the next day. Finally, the grayscale was analyzed by Image- J.

The cells were grouped and treated as described in section 2.8. The cells in each group were added with precooled RIPA and protease inhibitors, then the cells were fully lysed, centrifuged at 12000 rpm for 20 min at 4°C, and the protein concentration was determined by BCA method and quantified to 15 μg/10 μL. The latter steps were the same as above.

### 2.12 Cell immunofluorescence

Cells were uniformly seeded in 12-well plates with cell-specific slides placed in advance, and cells were grouped and treated as described in section 2.8. The cells were fixed with 4% paraformaldehyde for 30 min at room temperature and then incubated with 0.25%TritonX-100 for 10 min (membrane proteins) or 15 min (nuclear and cytoplasmic proteins) at room temperature. After that, the cells were blocked with BSA for 1 h, and the cells were covered with the next primary antibody solution and incubated at 4°C overnight: Iba-1 (1:100, Proteintech, China), CD86 (1:50, Boster, China), CD206 (1:400, Proteintech, China), TLR4 (1:50, Abclonal, China), p65 (1:100, Bioss, China), p-p65 (1:100, Bioss, China). The next day, the fluorescent secondary antibody (1:150, Bioss, China) was incubated in the dark for 1 h after rewarming at room temperature, and DAPI was incubated in the dark for 10 min after washing. Anti-fluorescence quencher was added to seal the slides, and the slides were observed under the fluorescence microscope and photographed under 20x objective lens with the same parameters as the control group. Finally, Image-J was used for analysis.

### 2.13 Statistical analysis

The results were compared using GraphPad Prism V8.0.2. The experimental data were represented as mea*n* ± SEM. The data were analyzed by one-way ANOVA followed by Tukey’s post-hoc tests for multiple comparisons. *p* < 0.05 was considered statistically significant.

## 3 Results

### 3.1 (+)-Borneol reduced hippocampal neuronal damage and apoptosis in SE mice

The expression of neuronal marker NeuN, pro-apoptotic protein Bax and anti-apoptotic protein Bcl-2 were examined in the hippocampus of SE mice.

**Figure 1:**
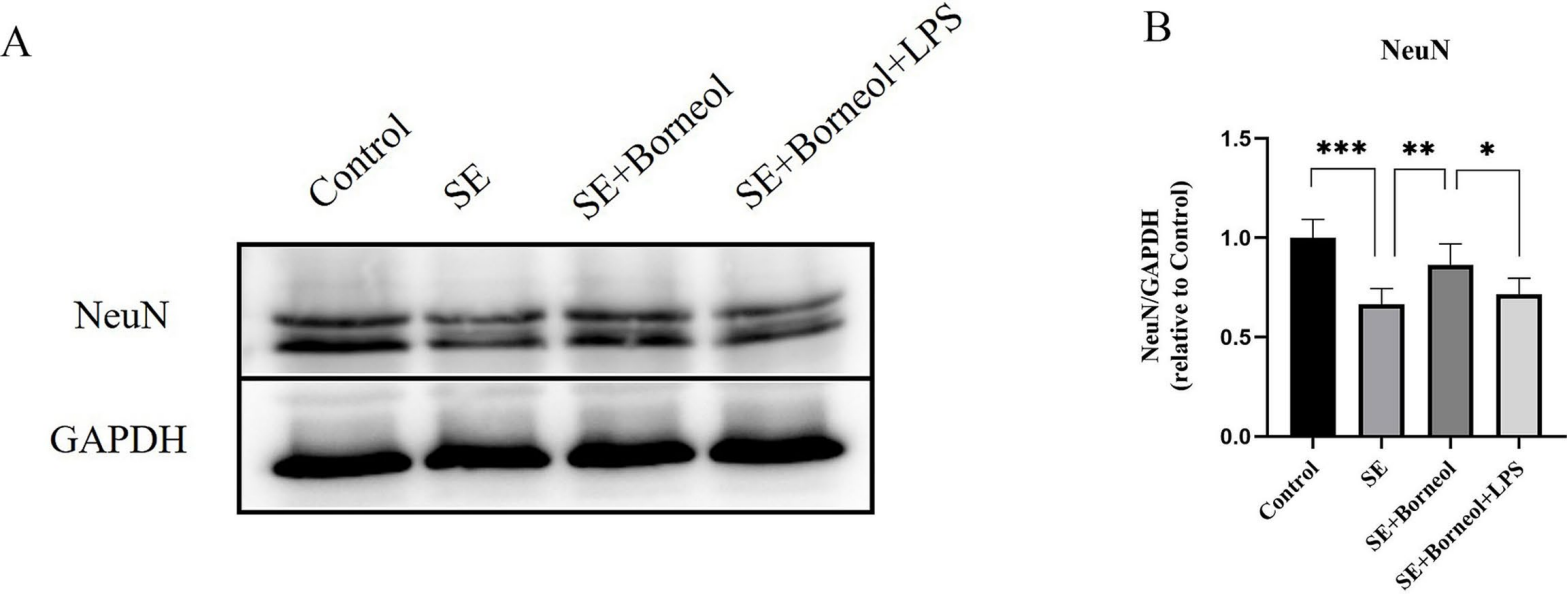
Effect of (+)-borneol on the NeuN expression in SE mice hippocampus. **(A)** Western-Blot representative diagrams of NeuN in each group of mice. **(B)** Analysis and statistics of Western-Blot results. ***p < 0.001; **p < 0.01; *p < 0.05; *n* = 6.

The results of Western Blot showed that the expression of NeuN in the SE group was significantly lower than that in the Control group (*p* < 0.001). (+)-Borneol treatment reduced hippocampal neuronal damage after SE (*p* < 0.01) and TLR4 activated by LPS partially reversed the effect (*p* < 0.05; Figures 1A,B).

**Figure 2:**
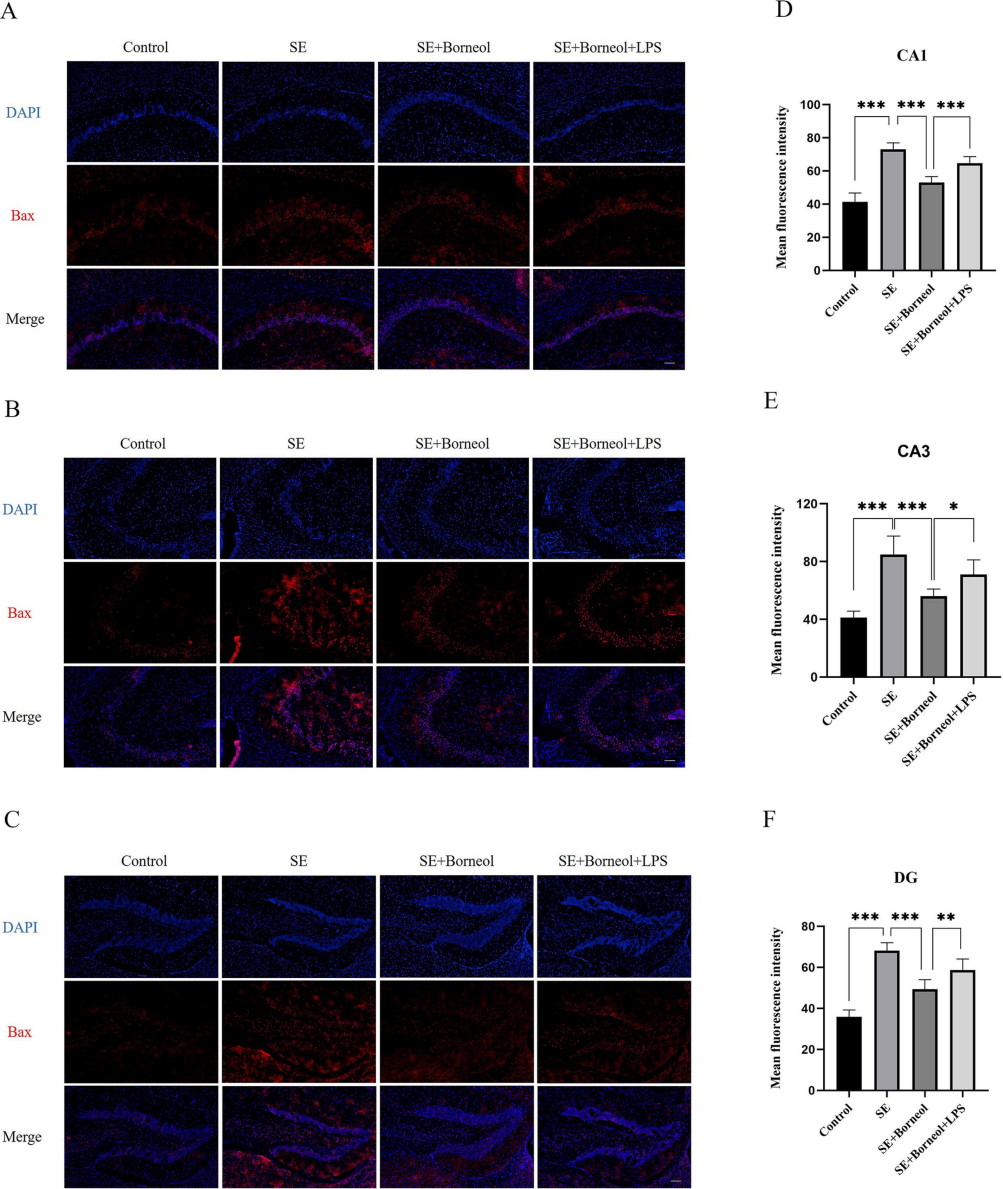
Effect of (+)-borneol on the Bax expression in SE mice hippocampus. **(A–C)** Immunofluorescence representative diagrams of Bax in CA1 **(A)**, CA3 **(B)**, and DG **(C)** region. **(D–F)** Analysis and statistics of immunofluorescence in each region. Bar = 200 μm. ***p < 0.001; **p < 0.01; *p < 0.05; *n* = 6.

**Figure 3:**
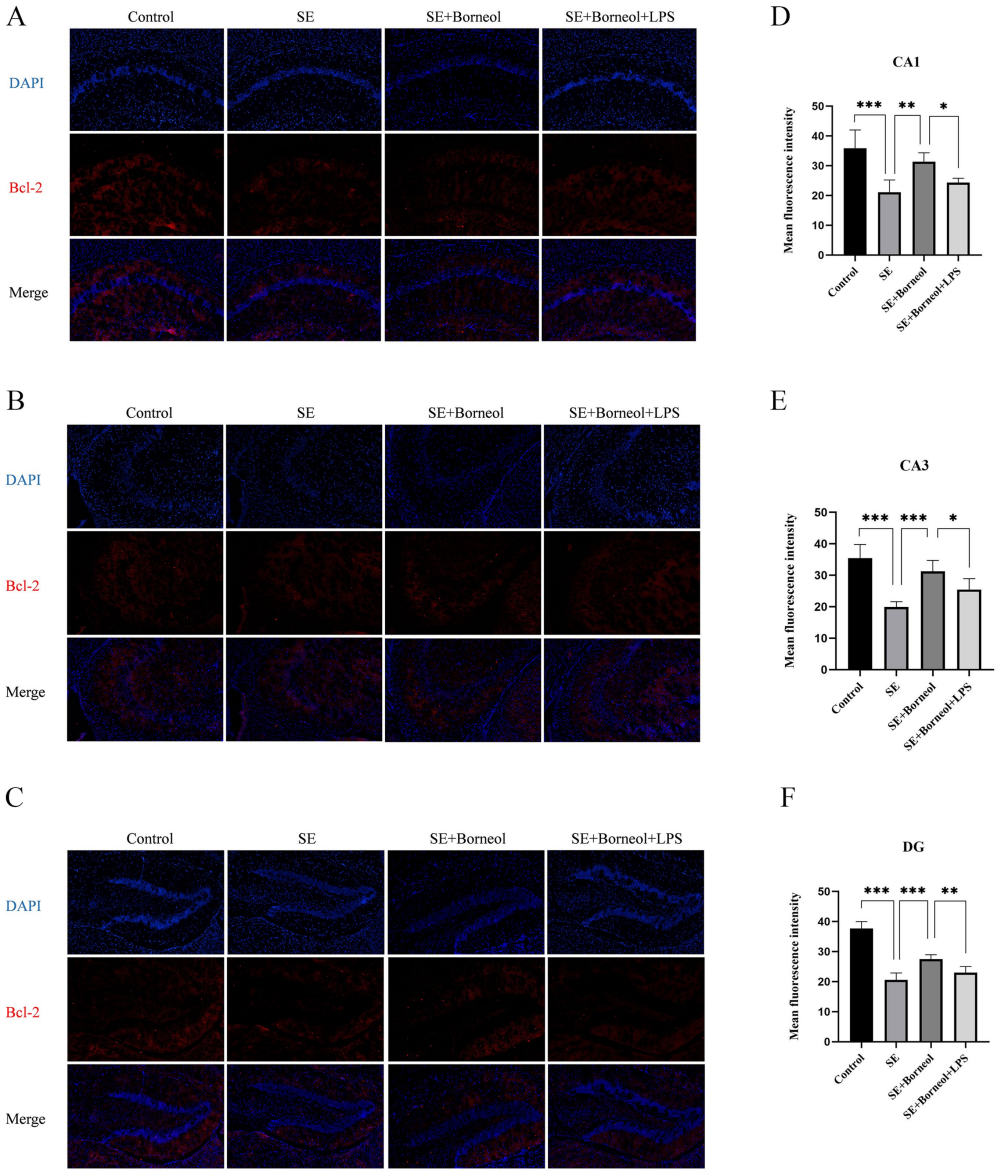
Effect of (+)-borneol on the Bcl-2 expression in SE mice hippocampus. **(A–C)**, Immunofluorescence representative diagrams of Bcl-2 in CA1 **(A)**, CA3 **(B)**, and DG **(C)** region. **(D–F)** Analysis and statistics of immunofluorescence in each region. Bar = 200 μm. ***p < 0.001; **p < 0.01; *p < 0.05; *n* = 6.

The results of immunofluorescence of Bax (Figure 2) and Bcl-2 (Figure 3) showed that the expression of Bax was increased (*p* < 0.001) and the expression of Bcl-2 was decreased (*p* < 0.001) in each brain region in the SE group compared with the Control group. Treatment with (+)-borneol reduced the expression of Bax (*p* < 0.001) and increased Bcl-2 (CA1, *p* < 0.01, CA3, DG *p* < 0.001). Activation of TLR4 reversed the anti-apoptosis effect of (+)-borneol (Bax, CA1 *p* < 0.001, CA3 *p* < 0.05, DG *p* < 0.01 and Bcl-2, CA1, CA3 *p* < 0.05, DG *p* < 0.01).

### 3.2 (+)-Borneol reduced microglia activation in the hippocampus of SE mice

The expression of microglia marker Iba-1 in the hippocampus of epileptic mice was detected.

**Figure 4:**
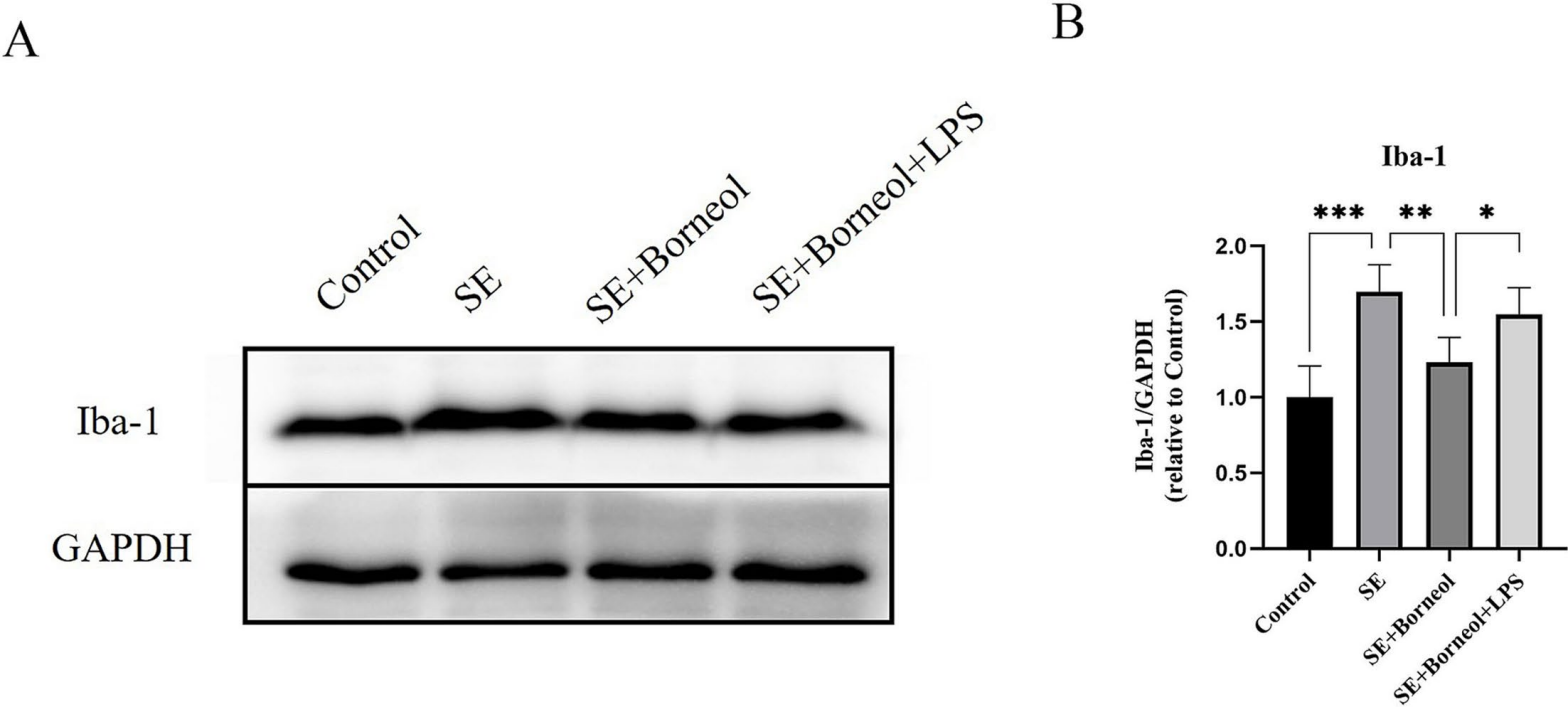
Effect of (+)-borneol on the Iba-1 expression in SE mice hippocampus. **(A)** Western-Blot representative diagrams of Iba-1 in each group of mice. **(B)** Analysis and statistics of Western-Blot results. ***p < 0.001; **p < 0.01; *p < 0.05; *n* = 6.

The results of Western Blot showed that the expression of Iba-1 in the SE group has a higher level than that in the Control group (*p* < 0.001). (+)-Borneol treatment reduced Iba-1 expression (*p* < 0.01) and TLR4 agonist LPS increased the Iba-1 expression compared with the SE + Borneol group (*p* < 0.05) (Figures 4A,B).

### 3.3 (+)-Borneol inhibited the activation of TLR4-NFκB signaling pathway in the hippocampus of SE mice

The expression of TLR4, p65/NFκB and p-p65/NFκB in the hippocampus of SE mice was detected.

**Figure 5:**
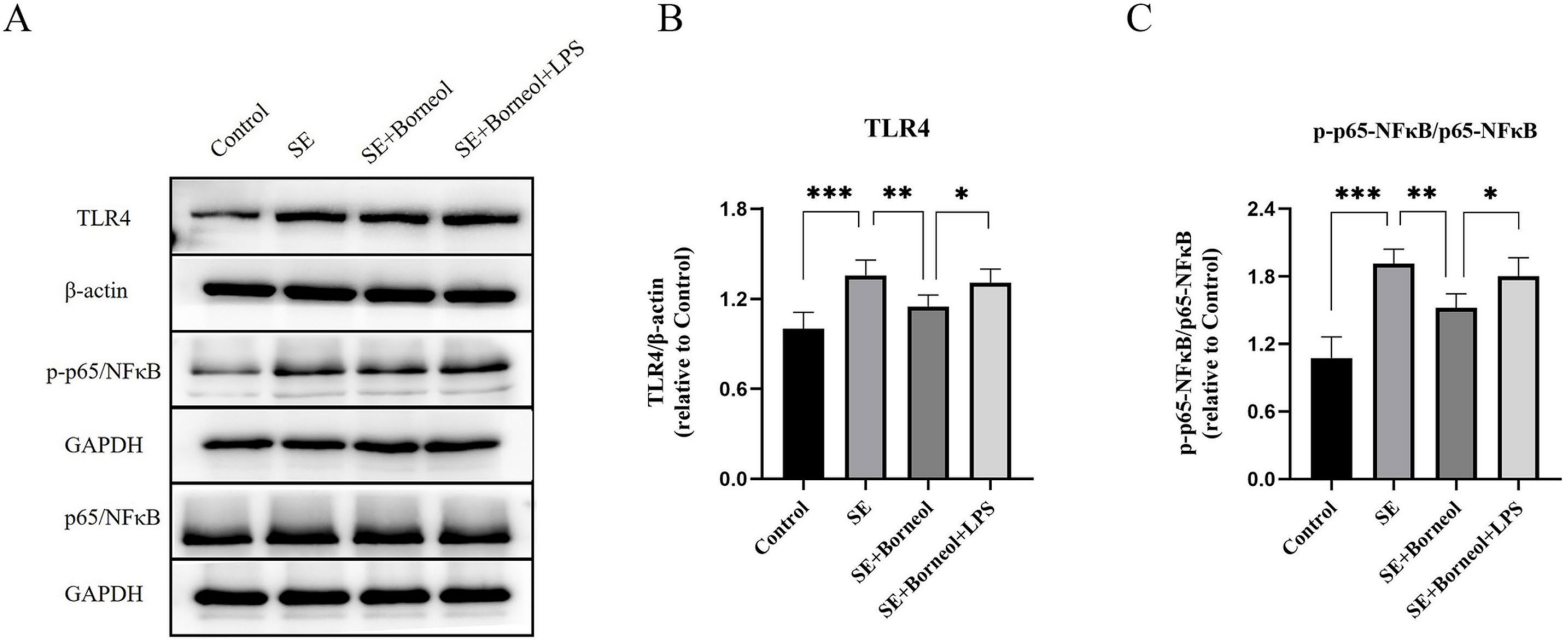
Effect of (+)-borneol on the TLR4, p65 and p-p65 expression in SE mice hippocampus. **(A)** Western-Blot representative diagrams of TLR4, p65 and p-p65 in each group of mice. **(B)** Analysis and statistics of TLR4 Western-Blot results. **(C)** Analysis and statistics of p-p65/p65 Western-Blot results. ***p < 0.001; **p < 0.01; *p < 0.05; *n* = 6.

Western Blot results showed that the expression of TLR4 and p-p65/p65 in the hippocampus of the SE group was significantly higher than that of the Control group (*p* < 0.001). After the treatment of (+)-borneol, the expression of TLR4 and pp65/p65 was decreased (*p* < 0.01). Compared with the SE + Borneol group, the SE + Borneol+LPS group had significant increases in the expression of TLR4 and p-p65/p65 (*p* < 0.05) (Figures 5A–C).

### 3.4 The optimal concentration of (+)-borneol in KA-induced BV2 microglia was 25 μM

**Figure 6:**
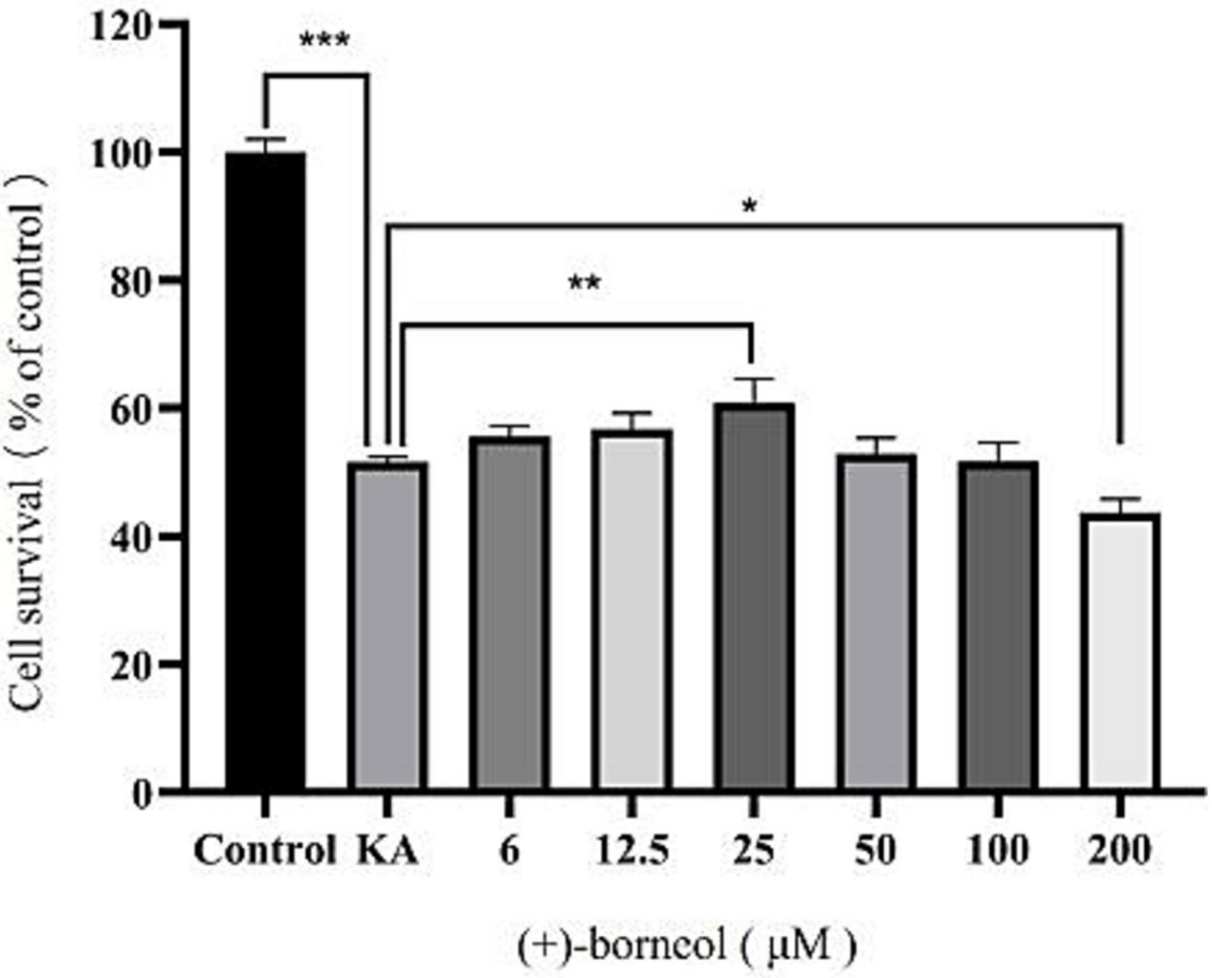
Different (+)-borneol concentration and microglia activity. ***p < 0.001; **p < 0.01; *p < 0.05; *n* = 3.

To determine the optimal concentration of (+)-borneol on KA-induced microglia, microglia were treated with different concentrations of (+)-borneol after 12 h of KA-induced, then co-cultured with KA for another 12 h. Cell viability was detected by CCK-8 (Figure 6). The results showed that treatment with 600 μM KA significantly decreased the viability of microglia (*p* < 0.001). The toxic effect of KA was counteracted by treatment with 25 μM (+)-borneol (*p* < 0.01).When the concentration increased to 200 μM, (+)-borneol produced toxic effects (*p* < 0.05). The concentration of (+)-borneol, which can antagonize the toxicity of KA and has high cell activity, was selected as the optimal concentration in this experiment, so it was determined to be 25 μM. (+)-Borneol inhibited KA-induced pro-inflammatory cytokines secretion in BV2 microglia.

### 3.5 (+)-Borneol inhibited KA-induced pro-inflammatory cytokines secretion in BV2 microglia

The levels of proinflammatory cytokines TNFα, IL-1β and anti-inflammatory cytokine IL-10 in the culture supernatant of BV2 microglia were detected by ELISA. The level of NO in the culture supernatant was detected by the Griess method.

**Figure 7:**
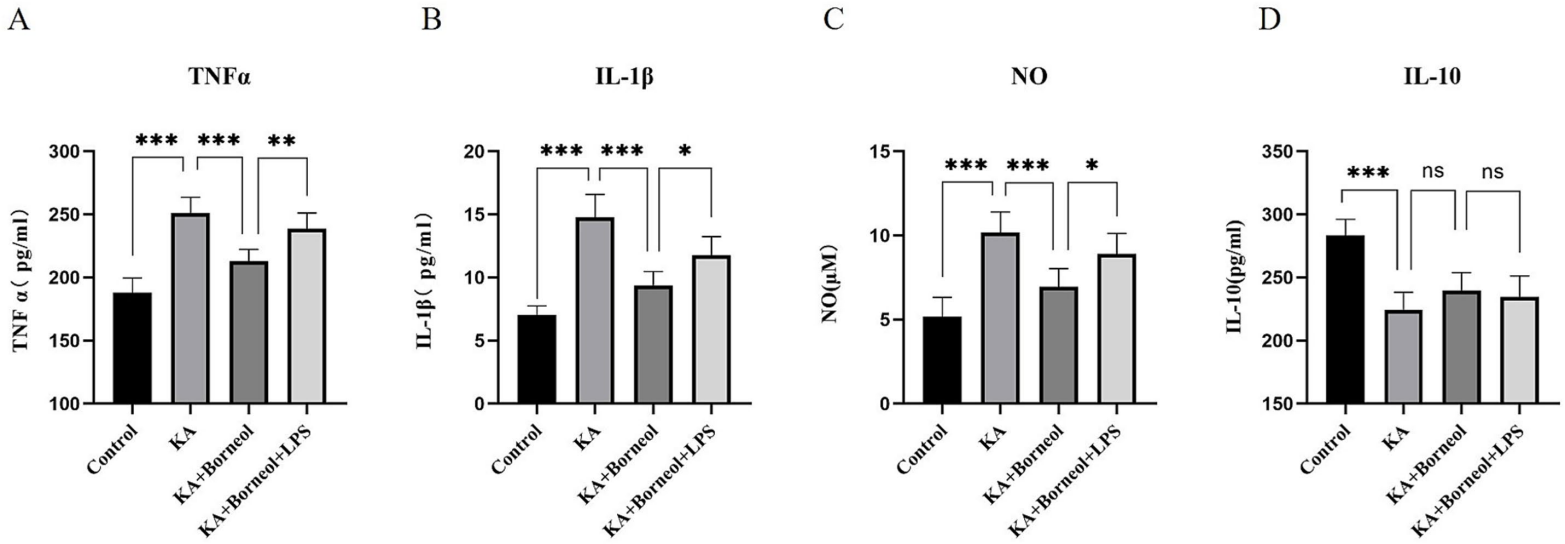
Effect of (+)-borneol on KA-induced inflammatory cytokine secretion in BV2 microglia. Statistical results of TNF-α **(A)**, IL-1β **(B)**, NO **(C),** and IL-10 **(D)** of BV2 microglia supernatant in each group. ***p<0.001; **p < 0.01; *p < 0.05; ns *p* > 0.05; *n* = 6.

Compared with the Control group, the secretion of three pro-inflammatory cytokines (TNFα, IL-1β and NO) was increased, and the secretion of anti-inflammatory cytokine (IL-10) was decreased in the KA group (*p* < 0.001). Compared with the KA group, the secretion of proinflammatory cytokines in the KA + Borneol group decreased (*p* < 0.001), and there was no significant difference in the secretion of anti-inflammatory cytokines (*p* > 0.05). Compared with the KA + Borneol group, the KA + Borneol+LPS group showed increased secretion of pro-inflammatory cytokines (TNFα *p* < 0.01, IL-1β and NO *p* < 0.05), but no significant difference in anti-inflammatory cytokines (*p* > 0.05) (Figure 7).

### 3.6 (+)-Borneol inhibited KA-induced activation of BV2 microglia and inhibited microglial polarization to M1 phenotype

Western Blot was used to detect the expression of Iba-1 in BV2 microglia. Immunofluorescence assay was used to detect the expression of Iba-1, M1 marker CD86 and M2 marker CD206 in BV2 microglia.

**Figure 8:**
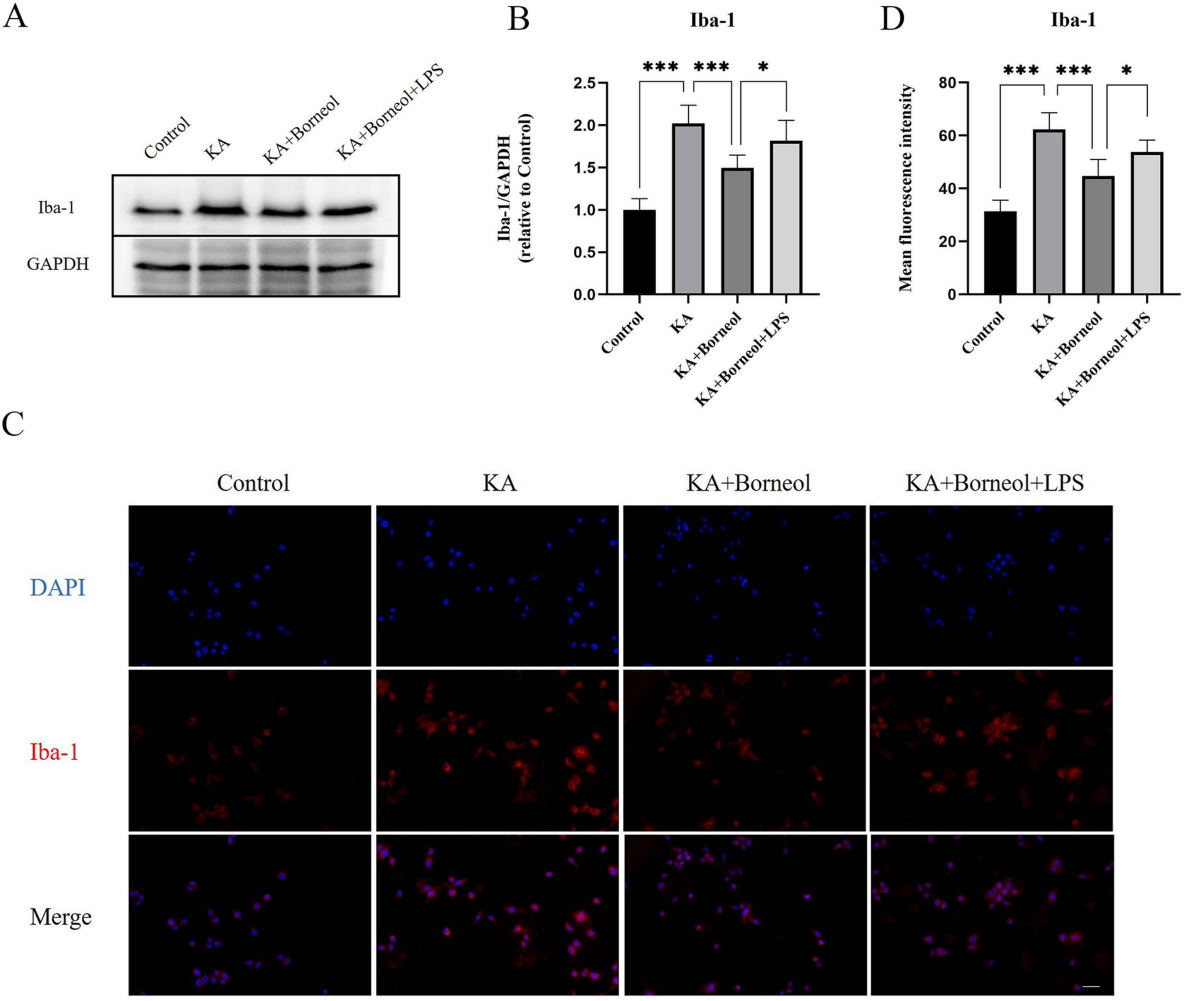
Effect of (+)-borneol on Iba-1 in KA-induced BV2 microglia. **(A)** Western-Blot representative diagrams of Iba-1 in each group of BV2 microglia. **(B)** Analysis and statistics of Western-Blot results. **(C)** Immunofluorescence representative diagrams of Iba-1 in each group of BV2 microglia. **(D)** Analysis and statistics of immunofluorescence results. Bar = 100 μm. ***p<0.001; **p < 0.01; *p < 0.05; ns *p* > 0.05; *n* = 6.

The expression of Iba-1 was detected by Western Blot (Figures 8A,B). Compared with the Control group, the expression of Iba-1 in the KA group increased (*p* < 0.001). Compared with the KA group, the KA + Borneol group had a significant reduction in the expression of Iba-1 (*p* < 0.001). Compared with the KA + Borneol group, the KA + Borneol+LPS group had a significant increase in the expression of Iba-1 (*p* < 0.05).

The results of immunofluorescence detection of Iba-1 showed that the average fluorescence intensity of Iba-1 in the KA group was higher than that in the Control group (*p* < 0.001). The average fluorescence intensity of Iba-1 in the KA + Borneol group was lower than that in the KA group (*p* < 0.001). Compared with the KA+ Borneol group, the KA + Borneol+LPS group had a significantly increased mean fluorescence intensity of Iba-1 (*p* < 0.05). The immunofluorescence results were identical to Western-Blot’s (Figures 8C,D).

**Figure 9:**
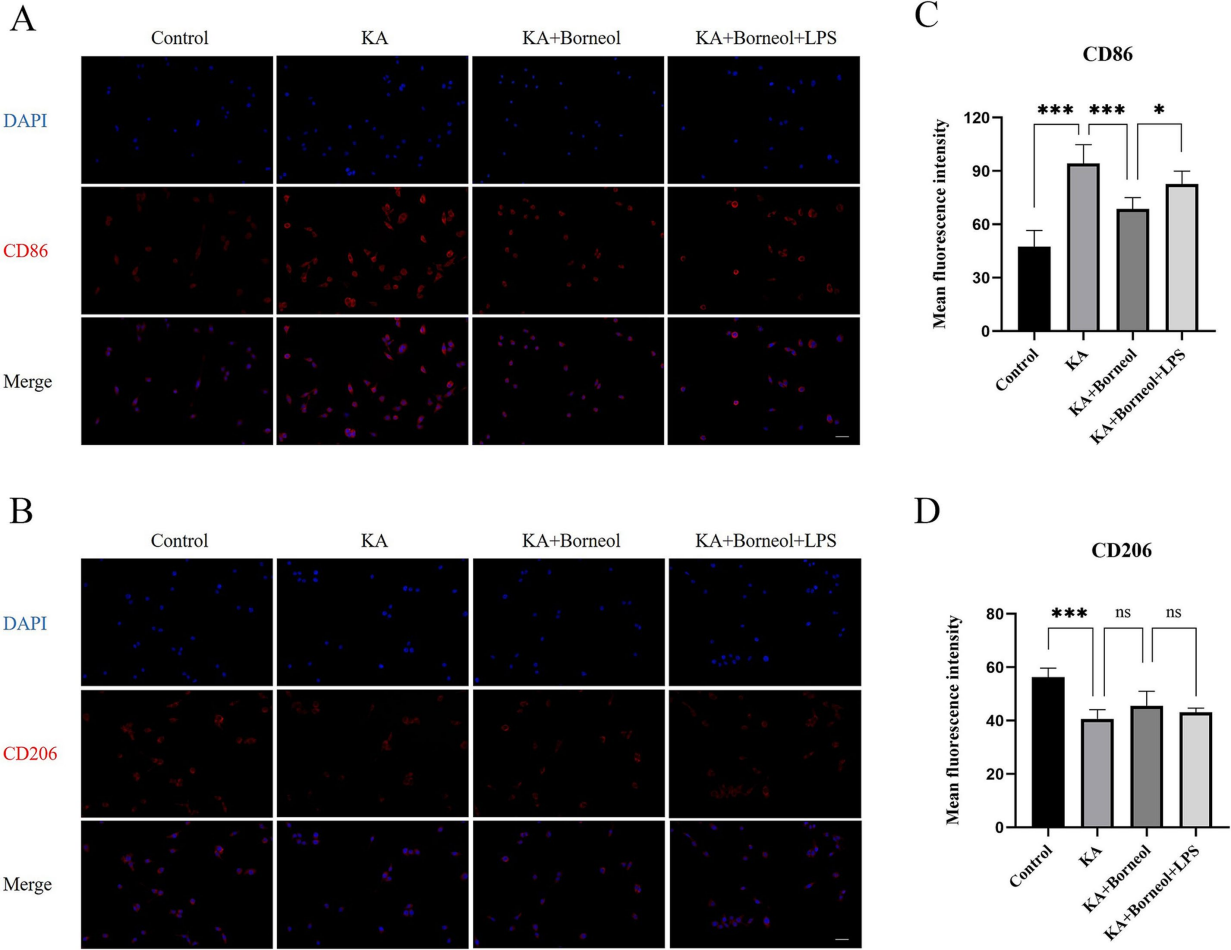
Effect of (+)-borneol on CD86 and CD206 in KA-induced BV2 microglia. **(A,B)** Immunofluorescence representative diagrams of CD86 **(A)** and CD206 **(B)** in each group of BV2 microglia. **(C,D)** Analysis and statistics of immunofluorescence results. Bar = 100 μm; ***p<0.001; **p < 0.01; *p < 0.05; ns *p* > 0.05; *n* = 6.

The results of immunofluorescence detection of CD86 and CD206 showed that, compared with the Control group, the average fluorescence intensity of CD86 in the KA group was increased (*p* < 0.001), and the average fluorescence intensity of CD206 was decreased (*p* < 0.001). Compared with the KA group, the average fluorescence intensity of CD86 in the KA + Borneol group was decreased (*p* < 0.001), and there was no significant difference in the average fluorescence intensity of CD206 (*p* > 0.05). Compared with the KA + Borneol group, the mean fluorescence intensity of CD86 was increased in the KA + Borneol+LPS group (*p* < 0.05), but there was no significant difference in CD206 (Figures 9A–D).

### 3.7 (+)-Borneol inhibited KA-induced activation of TLR4-NFκB signaling pathway in BV2 microglia

The expression of TLR4, p65/NFκB and p-p65/NFκB in each group were detected.

**Figure 10:**
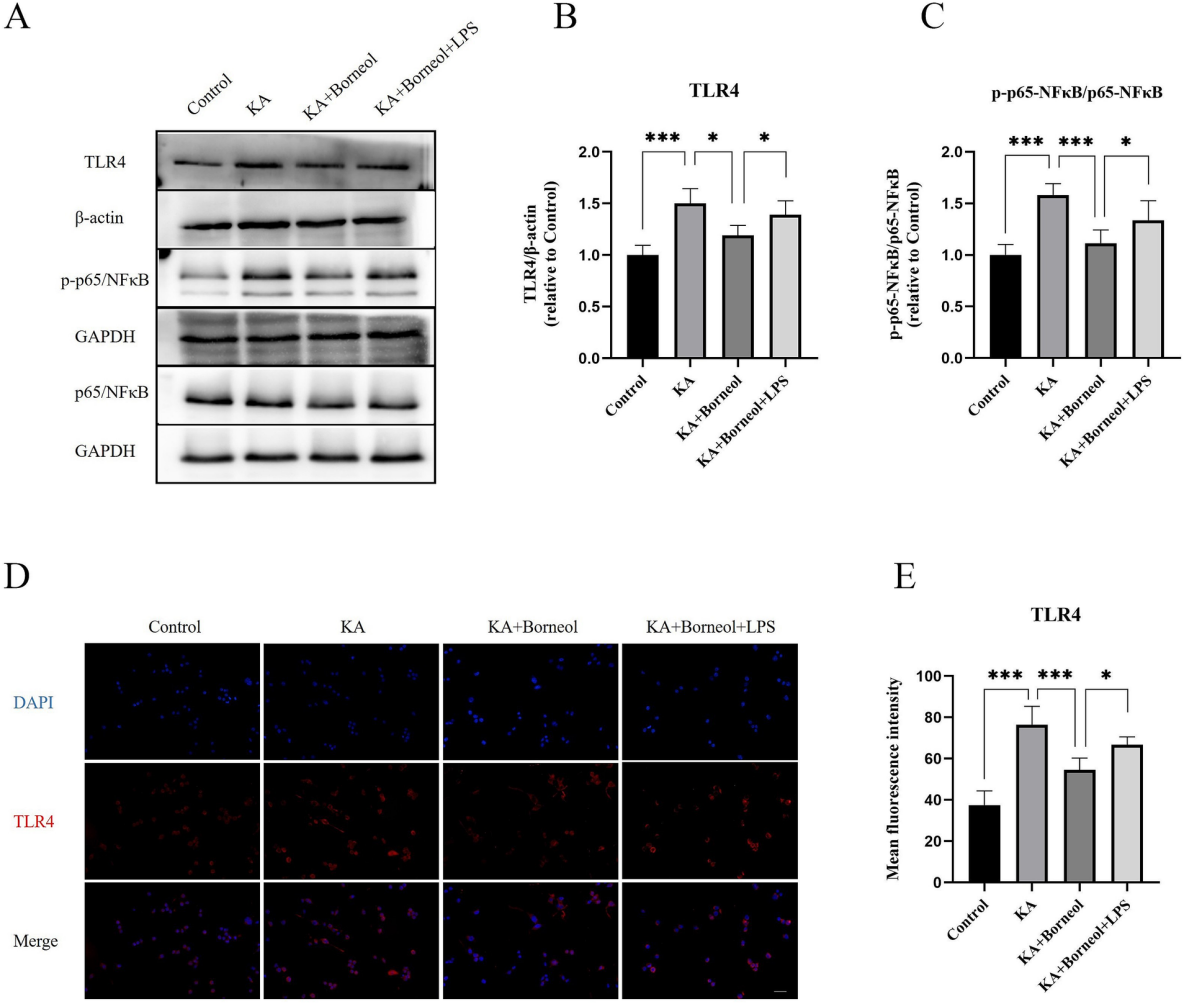
Effect of (+)-borneol on the TLR4, p65 and p-p65 expression KA-induced BV2 microglia. **(A)** Western-Blot representative diagrams of TLR4, p65 and p-p65 in each group of mice. **(B)** Analysis and statistics of TLR4 Western-Blot results. **(C)** Analysis and statistics of p-p65/p65 Western-Blot results. **(D)** Immunofluorescence representative diagrams of TLR4. **(E)** Analysis and statistics of immunofluorescence results. Bar=100 μm. ***p < 0.001, **p < 0.01, *p < 0.05, *n* = 6.

Western Blot results showed that the expression of TLR4 and p-p65/p65 in the KA group was significantly higher than that in the Control group (*p* < 0.001). The expression of TLR4 and pp65/p65 in KA + Borneol group decreased (TLR4 *p* < 0.05, p-p65/p65 *p* < 0.001). Compared with the KA + Borneol group, the KA + Borneol+LPS group had significantly increased expressions of TLR4 and p-p65/p65 (*p* < 0.05) (Figures 10A–C).

The results of TLR4 immunofluorescence showed that the mean fluorescence intensity of TLR4 in the KA group was increased compared with the Control group (*p* < 0.001). Compared with the KA group, the average fluorescence intensity of TLR4 in the KA + Borneol group was decreased (*p* < 0.001). Compared with the KA+ Borneol group, the KA + Borneol+LPS group had a significantly increased mean fluorescence intensity of TLR4 (*p* < 0.05). The immunofluorescence results were identical to those of Western Blot (Figures 10D,E).

## 4 Discussion

Our study demonstrated that (+)-borneol was neuroprotective in a pilocarpine-induced status epilepticus (SE) mouse model and inhibited KA-induced BV2 microglial neuroinflammation and M1 phenotype polarization.

According to the results, administration of (+)-borneol after SE reduced hippocampal neuronal damage and apoptosis, and inhibited microglial activation. TLR4 activation reversed the neuroprotective effects of (+)-borneol. In the microglia model induced by KA, (+)-borneol treatment reduced the secretion of pro-inflammatory cytokines, inhibited microglial activation and M1 phenotype polarization, and TLR4 agonist inhibited the anti-inflammatory effects of (+)-borneol. Moreover, (+)-borneol inhibited the activation of the TLR4-NFκB signaling pathway both *in vitro* and *in vivo*.

Currently, the most common clinically drug-resistant epilepsy to existing ASMs is mesial temporal lobe epilepsy (MTLE), which is also the most common type of focal epilepsy in adults (Vega-García et al., 2022; Panina et al., 2023). Studies on the mechanism of MTLE are based on animal models, and the pilocarpine-induced SE model is the most commonly used of these, which can produce similar pathological changes to human MTLE (Lévesque et al., 2021).

KA is an excitatory neurotransmitter glutamate analog that acts on *α*-amino3-hydroxy-5-methyl-4-isoxazolepropionic acid (AMPA) receptors to cause excessive Ca^2+^ influx. It can cause intracellular calcium overload and neuronal damage, resulting in epileptogenic effects (Zhang and Zhu, 2011). By acting on microglia, KA can simulate the epilepsy microenvironment, induce the proliferation and activation of microglia, and produce pro-inflammatory cytokines. Our previous experiment explored that the optimal injury concentration of KA on BV2 microglia was 600 μM, which was used as the model concentration for BV2 cells in this study.

Pathological changes such as abnormal increase of neuronal excitability in seizures and SE, neuroinflammation, oxidative stress, and mitochondrial dysfunction in epilepsy can lead to neuronal damage (Madireddy and Madireddy, 2023). The hippocampal neuron loss is the most obvious, which may develop into hippocampal damage, atrophy and sclerosis (Thompson, 2023). The loss of neurons and the resulting brain damage can lead to serious long-term consequences, such as impaired learning and memory, cognitive function, or the development of chronic epilepsy (Chepurnov et al., 2012).

Apoptosis is a form of programmed cell death which is closely related to neuronal damage in epilepsy (Henshall and Simon, 2005). Seizures can also activate the intrinsic apoptotic pathway of cells (Henshall, 2007). Bcl-2 family proteins regulate pro- and anti-apoptotic intracellular signals and are an important class of proteins that regulate apoptosis (Ashkenazi et al., 2017). Among them, anti-apoptotic protein Bcl-2 and pro-apoptotic protein Bax are important members. The anti-apoptotic and pro-apoptotic proteins of the Bcl-2 family maintain a dynamic balance under physiological effects, and promoting the positive or negative balance of apoptosis may become a new opportunity to intervene in diseases (Singh et al., 2019). In the present study, we also observed that (+)-borneol treatment increased the expression of NeuN and Bcl-2 and decreased the expression of Bax in the hippocampus. (+)-Borneol treatment reduced neuronal damage and apoptosis after SE, and TLR4 activation partially reversed the therapeutic effects of (+)-borneol.

Neuroinflammation mainly includes excessive activation of glia and excessive secretion of pro-inflammatory cytokines. Microglia can be activated in the early stage of epilepsy, phagocytose apoptotic cells, secrete appropriate amounts of inflammatory factors, and play a neuroprotective role (Hiragi et al., 2018). Subsequently, microglia secrete a large number of inflammatory cytokines, and excessive neuroinflammation induces astrocyte activation and dysfunction, reduces the seizure threshold, and mediates neuronal damage (Patel et al., 2019; Shen et al., 2023; Henning et al., 2023). Seizures further aggravate neuroinflammation, forming a vicious cycle and accelerating the process of epilepsy (Pracucci et al., 2021).

Our previous study has shown that microglia and astrocytes are rapidly activated and the secretion of inflammatory cytokines is increased after SE in rats (Gao et al., 2023; Tian et al., 2019). (+)-Borneol reduces the secretion of pro-inflammatory cytokines in the hippocampus of rats with SE. However, the effect of (+)-borneol on the secretion of pro-inflammatory and anti-inflammatory cytokines in microglia has not been investigated *in vitro*. Our results suggest that (+)-borneol inhibits microglia overactivation both *in vivo* and *in vitro*. *In vitro*, (+)-borneol treatment reduced KA-induced secretion of pro-inflammatory cytokines but had no significant effect on anti-inflammatory cytokines in BV2 microglia. TLR4 agonist treatment could partially reverse the anti-inflammatory effects of (+)-borneol. In addition, (+)-borneol has been reported to ameliorate epileptic seizures by inhibiting the excitability of glutamatergic synaptic transmission. These results suggest that (+)-borneol, as an anti-inflammatory agent, may have a therapeutic effect on epilepsy modification.

In recent years, microglia have been divided into two phenotypes according to their functions: the pro-inflammatory neurotoxic M1 and the anti-inflammatory neuroprotective M2. These two phenotypes are in dynamic changes and can be transformed into each other (Kwon and Koh, 2020). The regulation of M1 and M2 phenotype polarization has become an effective target for a variety of drugs to inhibit neuroinflammation and a potential direction for the treatment of nervous system diseases (Zhang et al., 2019; Jiang et al., 2021; Long et al., 2024). Previous studies have reported that targeting microglial phenotype polarization in epilepsy can reduce neuronal damage (Peng et al., 2019), and activation of M1 microglia can promote SE-induced neuroinflammation (Liang et al., 2023). The results of our study showed that (+)-borneol inhibited KA-induced M1 but not M2 polarization of BV2 microglia, suggesting that (+)-borneol inhibits the proinflammatory phenotype of microglia polarization.

TLR4 is a transmembrane protein that recognizes exogenous ligand LPS and endogenous ligands produced by stress and cell injury (Paudel et al., 2020). After binding to the ligand, it can activate the downstream NF-κB signaling pathway, secrete and release inflammatory factors, and activate the inflammatory cascade (Shi et al., 2018). TLR4 inhibitors can effectively improve the symptoms of epilepsy in mice (Dong et al., 2022).

NF-κB is a widely expressed transcription factor that is associated with biological processes of neuroinflammation and apoptosis (Dresselhaus and Meffert, 2019; Shih et al., 2015). P65 (Rel A) is a member of the NF-κB family. Under physiological conditions, p65 is mainly distributed in the cytoplasm in the form of p50-p65 heterodimers, which bind to IκB inhibitory proteins. Under pathological stimulation, IκB undergoes phosphorylation and degradation, releasing p65 into the nucleus. The phosphorylation of P65 can promote the binding of p65 to the nucleus, initiate the transcription of inflammation-related genes, and promote the occurrence of neuroinflammation (Aloor et al., 2015).

After binding to ligands, TLR4 distributed on the membrane of microglia can activate the downstream NF-κB, promote its nuclear translocation, promote the polarization of microglia to M1 phenotype, and secrete a large number of proinflammatory cytokines (Li et al., 2022; Younger et al., 2019). Previous studies have shown that (+)-borneol inhibits the activation of the NF-κB signaling pathway in mice with sepsis and in microglia and ischemic stroke models (Wang et al., 2019; Chang et al., 2017), but the effect of (+)-borneol on its upstream TLR4 has not been investigated. The present study found that (+)-borneol reduced TLR4 expression and p-p65/p65 ratio in the hippocampus of SE mice and KA-induced BV2 microglia. The effect of (+)-borneol was partially reversed by administration of TLR4 agonist LPS, suggesting that the effects of (+)-borneol on microglia neuroinflammation and phenotype polarization may be mediated by targeting TLR4 and inhibiting TLR4-NFκB signaling pathway.

The results of this study suggest that (+)-borneol can inhibit neuroinflammation in epilepsy and may be used as a potential anti-epileptogenesis drug for epilepsy modifying therapy, delaying the progression of epilepsy and improving the long-term prognosis of epilepsy.

However, the present study only investigated the pathophysiological changes of (+)-borneol in SE mice within 7 days and the possible mechanisms, but not the role of (+)-borneol in chronic epilepsy. Further studies are needed to investigate the electrophysiological effects of (+)-borneol on SE by monitoring the frequency and duration of chronic and recurrent seizures with electroencephalogram. It should be mentioned that as the data of this study represents the results in male mice only, conclusions are limited to males.

Overall, data from our study suggests that (+)-borneol alleviates neuroinflammation and inhibits microglia M1 phenotype polarization during epilepsy both *in vitro* and *in vivo*.

## Data Availability

The raw data supporting the conclusions of this article will be made available by the authors, without undue reservation.
