# Supplementary figures and images for: (+)-Borneol inhibits neuroinflammation and M1 phenotype polarization of microglia in epileptogenesis through the TLR4-NFκB signaling pathway
Source: Front Neurosci. 2024 Nov 13;18:1497102. doi: 10.3389/fnins.2024.1497102 (PMC11599196; doi:10.3389/fnins.2024.1497102)

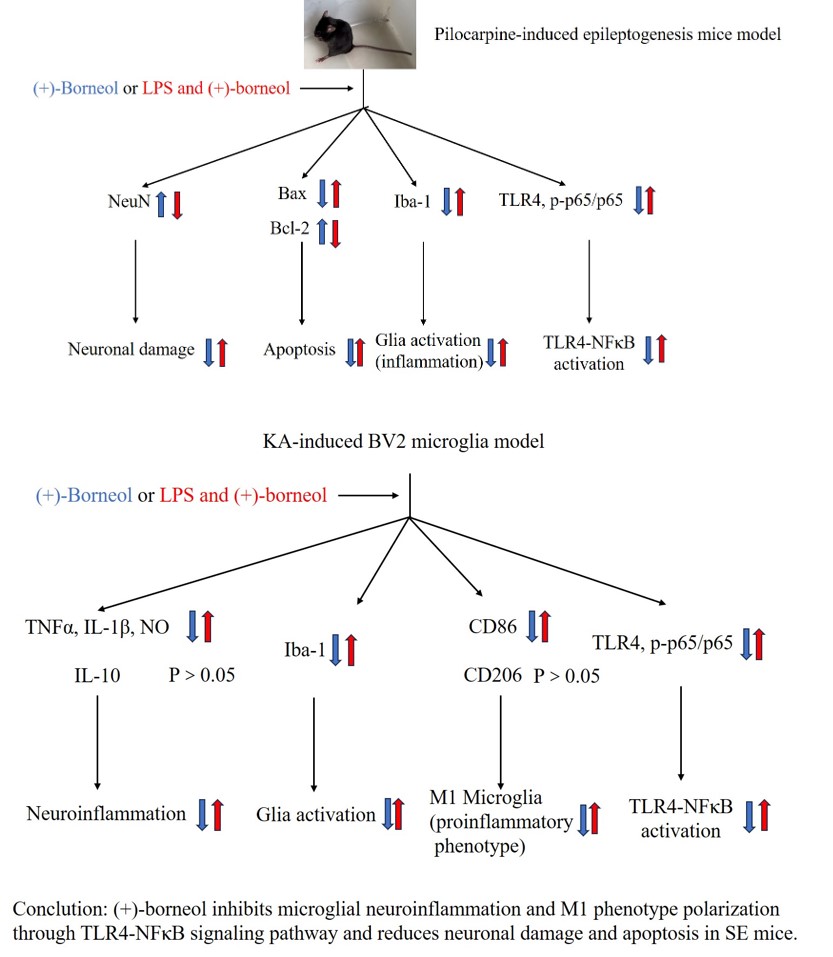

Supplement: Supplementary file 1 [file Image_1.jpeg]
